# Sapotaceae Family Fruits from Central America: Botanical, Phytochemical and Nutraceutical Insights—A Review

**DOI:** 10.3390/plants14213297

**Published:** 2025-10-29

**Authors:** Zaira Guadalupe Ibarra-Manzanares, Alayla Guadalupe Ibarra-Manzanares, Lluvia de Abril Alexandra Soriano-Melgar, Martha Monzerrath Orozco-Sifuentes, Jesús Andrés Salas-Tovar, Sarahí del Carmen Rangel-Ortega, Raúl Rodríguez-Herrera

**Affiliations:** 1 School of Chemistry, Autonomous University of Coahuila, Saltillo 25280, Mexico; zairaibarra@uadec.edu.mx (Z.G.I.-M.); alayla_ibarra@uadec.edu.mx (A.G.I.-M.); m-orozco@uadec.edu.mx (M.M.O.-S.); andres_salas@uadec.edu.mx (J.A.S.-T.); 2 Researchers for Mexico (IIxM-SECIHTI) Commissioned by the School of Chemistry, Autonomous University of Coahuila, Saltillo 25280, Mexico; lluviasoriano_fcq@uadec.edu.mx; 3 Department of Food Science and Technology, Antonio Narro Autonomous Agricultural University, Saltillo 25315, Mexico; sarahi.rangel@uaaan.edu.mx

**Keywords:** bioactive compounds, tropical fruit, biological properties, natural products

## Abstract

The Sapotaceae family includes various fruit species of ecological, economic, and nutritional importance, among which *Pouteria sapota* (mamey sapote), *Manilkara zapota* (zapote chico), *Pouteria campechiana* (canistel), and *Pouteria viridis* (zapote verde) stand out, widely distributed throughout Mesoamerica. These species have traditionally been used as a source of food, natural medicine, and other products of cultural value. In recent decades, there has been growing scientific interest in studying their phytochemical composition, which has led to the identification of important secondary metabolites such as phenolic compounds, carotenoids, flavonoids, and triterpenes, associated with beneficial health effects. This article summarizes and analyzes the available information on their diversity, traditional use, chemical composition, and biological activities. It also highlights research opportunities aimed at the development of functional products, therapeutic applications, and nutraceuticals, as well as the sustainable use of these species.

## 1. Introduction

Fruit species from the Sapotaceae family represent a significant component of Mesoamerican biodiversity, not only due to their nutritional value but also because of the range of bioactive compounds that they contain, which confer functional properties of interest for human health [1,2]. *Pouteria sapota*, *Manilkara zapota*, *Pouteria campechiana*, and *Pouteria viridis* are considered priority species in Mexico due to their central role in the diet, traditional medicine, and cultural practices of Indigenous and rural communities, as well as for their economic importance. Since pre-Hispanic times, the fruits, seeds, bark, and derived products from these species have been used in diet, preparation of medicinal products, and ethnobotanical applications [3,4].

Sapotaceae fruits are characterized by their complex nutritional profile, which includes carbohydrates, dietary fiber, proteins, lipids, vitamins, and minerals. Specifically, *Manilkara zapota* and *Pouteria campechiana* differ in their composition, reflecting interspecific variations and changes related to the ripening process, such as increases in soluble sugars and decreases in fiber and organic acids during ripening [5,6]. In addition, these fruits contain vitamins such as ascorbic acid and bioactive pigments such as carotenoids and lycopene, which contribute to their antioxidant activity and the regulation of physiological processes important for animal health [7,8,9]

The phytochemical composition of these species is extensive and includes flavonoids, tannins, phenols, alkaloids, saponins, quinones, and triterpenes, which are distributed in various tissues such as pulp, seed, leaf, and bark [10,11]. The extraction of these compounds depends on appropriate techniques, which take in account the type of solvent, the preparation of plant material, and assisted technologies such as ultrasound and homogenization, which improve yields and maintain the biological activity of the identified metabolites [12,13].

Scientific interest in these species has grown over the last decade, thanks to the identification of secondary metabolites and the biological activities attributed to these compounds, including antioxidant, anti-inflammatory, immunomodulatory, antimicrobial, antifungal, neuroprotective, and cytotoxic properties [14,15,16,17]. Both hydrophilic and lipophilic compounds contribute to these properties, modulating the activity of enzymes and inflammatory mediators and cell signaling pathways, which confer them therapeutic potential against metabolic, neurodegenerative, infectious, and oncological diseases [18,19,20].

Thanks to their phytochemical and functional profile, fruits and by-products of Sapotaceae have attracted interest in the food and pharmaceutical industries, especially for the development of functional products, nutraceutical supplements, and bioactive agents with therapeutic applications. Their pharmacological potential includes antihyperlipidemic, antidiabetic, anti-inflammatory, antimicrobial, and neuroprotective effects, positioning these species as sustainable resources of bioactive compounds with applications in human health [21,22].

In this context, this review critically examines the available literature on the diversity of the chemical composition, biological activities, and industrial applications of Sapotaceae species, highlighting their relevance as a valuable natural resource and justifying research related to their conservation, characterization, and use, as well as the design of strategies to maximize their nutritional and therapeutic benefits.

## 2. Data Collection and Search Strategy

The information used in this review was obtained through a systematic literature search of specialized databases, including Scopus, Web of Science, ScienceDirect, PubMed, SpringerLink, and MDPI, covering the period 1990–2025. Combinations of keywords related to the Sapotaceae family and its main genera (*Pouteria* and *Manilkara*) were used, along with terms linked to taxonomy, distribution, domestication, phytochemical composition, and biological activities.

Only peer-reviewed sources and documents with verifiable information were considered, excluding duplicate reports or those without methodological support. The selected species (*Pouteria sapota*, *Manilkara zapota*, *Pouteria campechiana*, and *Pouteria viridis*) were prioritized for their biocultural relevance in Mesoamerica, their pre-Columbian domestication, and the availability of taxonomic and phytochemical studies.

This strategy allowed for the integration of the most recent and reliable scientific evidence on diversity, chemical composition, and functional potential of the most representative species of the Sapotaceae family.

## 3. Botanical Classification and Geographical Distribution

Taxonomy makes it possible to understand the evolutionary relationships between genera and species, while geographic distribution provides information on the origin, domestication, and expansion of plants in different habitats. The above is important to assess the relevance of the different plant families, as well as their conservation, utilization, and possible biotechnological applications. In this case, the Sapotaceae family is distinguished by its great ecological, economic, and cultural significance, mainly in tropical regions, where it has been domesticated since pre-Hispanic times. However, its taxonomy has been very complex because there is a high degree of synonymy; that is, the same species has been described on different occasions, where it has been given different names. Additionally, knowledge of morphological similarities, known as homoplasy, and characters shared with other species that come from a common ancestor, called synapomorphies, is not clear for the Sapotaceae family. This has led to molecular and genomic studies being used to complement the classical morphological approaches to clarify the classification of this family [1,2,23,24].

### 3.1. Botanical Classification

**Figure 1:**
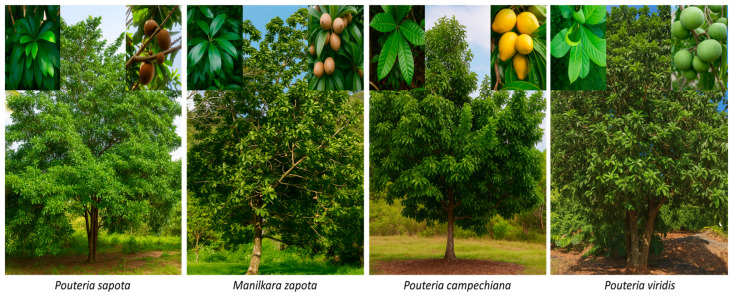
Representative trees and fruits of native fruit species of the Sapotaceae family from Mesoamerica, showing characteristic features of foliage and fruit morphology at different stages of ripening.

The *Sapotaceae* Juss. (1789) family, belongs to the order Ericales and comprises around 53 to 70 genera and between 1100 to 1300 species of trees and shrubs [2,25,26,27]. Most members of this family are characterized by the presence of laticifers with milky latex of white color, occasionally yellowish to greenish, with simple entire leaves, hermaphroditic flowers, pentamerous with numerical variations, fleshy fruits of the berry type or on rare occasions of the drupe type, with seeds generally considered large, smooth, and shiny [28,29,30]. The internal classification of the family has been variable over time. Pennington TD [31] proposed five tribes (*Chrysophylleae*, *Isonandreae*, *Omphalocarpeae*, *Mimusopeae*, and *Sideroxyleae*). However, molecular studies involving nuclear and plastid DNA [32,33,34] allowed the recognition of three subfamilies *Chrysophylloideae*, *Sapotoideae*, and *Sarcospermatoideae* [24,35], which was later confirmed by other studies based on genomic analyses with more recent techniques [36,37,38]. Among the most representative genera, *Pouteria* can be mentioned, which is considered one of the most diverse and taxonomically most complex, which led to the recognition of between 200 to 325 species, in addition to being considered the most polyphyletic genus and subject to a large number of segregations (*Beccariella*, *Planchonella*, *Sersalisia*, *Van-royena*) [39,40]. This genus includes species of great economic and cultural interest such as *Pouteria sapota* (mamey) and *P. campechiana* (canistel), followed by *Manilkara*, which has around 78 species distributed almost worldwide, standing out for the production of latex used in the manufacture of chewing gum and various edible fruits, among them *Manilkara zapota*, known as chicozapote, which is one of the most relevant species of this genus [41,42,43,44]. Also, the *Chrysophyllum* genus can be mentioned, which is represented in Mesoamerica by *Chrysophyllum cainito* (caimito, also known as star apple), which is highly valued for its sweet pulp and even more appreciated for the bioactive properties of its fruits [45]. Therefore, recent advances in molecular systematics and phylogenomics have clarified evolutionary relationships, identifying three main clades and useful molecular markers, such as the loss of the *ndhf* gene in *Pouteria* species [36,38]. Altogether, the Sapotaceae family constitutes a taxonomically complex group, where the genera *Pouteria* and *Manilkara* occupy a central role in the Neotropics and in Mesoamerica, not only because of their ecological importance, but also for their agri-food and cultural relevance since pre-Hispanic times (Figure 1).

### 3.2. Geographical Distribution

**Figure 2:**
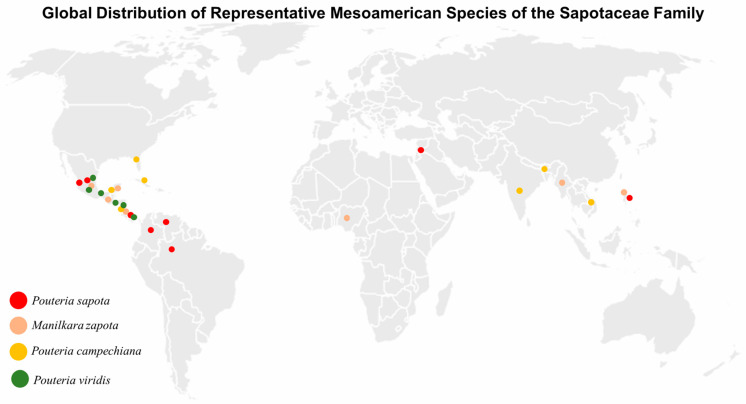
Global distribution of four domesticated Mesoamerican Sapotaceae species. Colored dots represent *Pouteria sapota*, *Manilkara zapota*, *P. campechiana*, and *P. viridis* distribution, showing native centers in Mesoamerica and introduced populations in tropical regions worldwide.

The Sapotaceae family presents a pantropical distribution (Figure 2); that is, it is in all tropical regions of all continents or of the entire world, with a center of diversity mainly in America, followed by Africa and Southeast Asia [23,46]. However, it has been reported that the greatest concentrations are found mainly in the Amazon, the Brazilian Atlantic Forest, and Mesoamerica [47]. However, there are plants of this family that are very well adapted to arid and semi-arid environments, such as *Argania spinosa* in Morocco or some species of *Sideroxylon* in the Caribbean and Mexico [39]. In Mesoamerica and the Caribbean, the *Pouteria*, *Chrysophyllum*, and *Manilkara* genera have not only served as structural elements of forests, but have also been used as food, medicinal, and cultural resources since pre-Hispanic times [41,45]. Among these, *Pouteria sapota* (mamey), *Pouteria campechiana* (canistel), *Manilkara zapota* (chicozapote), and *Chrysophyllum cainito* (caimito) are examples of domesticated species, widely cultivated throughout the world [31,46]. It has been reported that the natural dispersal of Sapotaceae has been closely linked to frugivorous fauna, while their expansion outside the centers of origin has been mainly favored by human action, through the domestication of fruit trees [45,48].

#### 3.2.1. *Pouteria sapota* (Mamey Sapote)

The mamey sapote (*Pouteria sapota*) is native to Mexican Southern and Southeastern and the lowlands of Central America, where it has been domesticated and cultivated since pre-Columbian times [49,50,51]. Mexico constitutes a center of diversity for the Sapotaceae species, especially in the Yucatán Peninsula and in the central-western region of Michoacán, where high morphological variability in fruits (shape, size, texture, and aroma of the pulp) is observed, associated with contrasting environmental conditions [43,52]. Its natural habitat corresponds to tropical and subtropical evergreen forests, at altitudes from 0 to 1300 m.a.s.l., its range covers Mexico’s Atlantic slope from Veracruz to Tabasco and the Pacific slope from Jalisco to Chiapas; it further extends southward across Central America, reaching as far as Panama [45,46]. Currently, cultivation has expanded to other tropical regions of the world, including the Caribbean, South America, Florida in the United States of America, the Philippines, Vietnam, Malaysia, Israel, Spain, and Australia [50,51]. Mamey thrives in warm and humid climates with abundant rainfall [climates Aw, Am, and A(C)(m)], which has allowed the identification of three differentiated gene groups with importance for breeding programs [2,50]. Mexico is considered the main Sapotaceae producer. In 2014, 1651 ha were cultivated in 15 different Mexican states, with 17,586 tons of fruit production, highlighting production in Mexican states such as Yucatán, Guerrero, Chiapas, and Michoacán [50]. Its red and aromatic pulp is consumed fresh or processed, whose processing includes the production of beverages, desserts, and ice creams, which makes it a key resource both in family orchards and in commercial agroforestry systems. Its growing international demand reflects its nutraceutical value and the global interest in exotic fruits [49,53,54].

#### 3.2.2. *Manilkara zapota* (Sapodilla, Chicozapote)

The chicozapote (*Manilkara zapota*) is native to southeastern Mexico, Belize, and Guatemala, especially in the Yucatán Peninsula, where it has been domesticated since pre-Hispanic times for its sweet fruit and the latex of its bark, used in the manufacture of chewing gum [42,55]. Its natural distribution extends to Honduras and Nicaragua, in tropical sub-evergreen forests and medium sub-humid jungles, mainly at altitudes of 0 to 800 m.a.s.l. [45]. Historically, latex exploitation reached great economic relevance in the Mexican Caribbean, especially in Quintana Roo during the first half of the 20th century. Currently, its importance lies on the production of fresh fruits and in its nutraceutical potential, given its richness in polyphenols and carotenoids [27,53,56]. In Mexico, it is mainly concentrated in Campeche, Quintana Roo, Yucatán, Tabasco, and Chiapas, although it is also reported in Veracruz and Oaxaca [50]. In 2014, more than 4800 ha were recorded as cultivated in the Yucatán Peninsula, considered the center of origin and genetic diversity of this specie. Its global dispersion is associated with the Spanish colonization, which introduced this species to the Philippines and from there to India, Sri Lanka, Thailand, Malaysia, and West Africa, regions where it is now extensively cultivated [57]. Taxonomically, it presents a wide synonymy (*Achras zapota*, *Calocarpum mammosum*, *Lucuma mammosa*, *Manilkara achras*), although the accepted name is *Manilkara zapota* (L.) P. Royen [56,58]. The tree can reach up to 45 m in height and produce between 2000 and 4000 fruits annually, as well as 2 to 10 kg of latex [59], although this value usually varies widely according to age, climate, management, among others.

#### 3.2.3. *Pouteria campechiana* (Canistel, Yellow Sapote)

The canistel (*Pouteria campechiana*), also known as yellow sapote, is native to Mesoamerica, with a natural distribution from Mexico to Panama [46,60]. Generally cultivated in yards and family orchards, it is rarely found in the wild. It has been introduced and naturalized in Cuba, Florida, the Philippines, India, and Bangladesh [5]. The tree reaches 12 to 25 m in height and produces fruits with a hard-yellow skin (hence its name yellow sapote) and dry, sweet pulp, of a tone or color similar to egg yolk [60]. Traditionally, its fruits are consumed fresh, but they are also found in different forms as processed products, including industrial applications such as flour and even used for biodiesel production [61]. Additionally, it is a multipurpose tree, where its wood is used in construction and its leaves and seeds are mainly used in traditional medicine due to their bioactive compounds with antioxidant, antibiotic, and anti-inflammatory properties [60,62].

#### 3.2.4. *Pouteria viridis* (Green Sapote)

The green sapote (*Pouteria viridis*) is considered native to the highlands of Mexico and Guatemala, and its distribution has been observed as far as Costa Rica [46,55,63]. It is a plant that grows mainly in humid mountainous environments, where it has been reported to grow between 1000 and 1500 m in Guatemala, and between 100 and 400 m in Nicaragua and Costa Rica [64]. In general, this specie is associated with highlands, so lower values than the previously mentioned altitudes may be due to local humid microclimates [63]. This species is cultivated especially in family orchards in Guatemala and El Salvador, where it is part of the traditional diet and local agroecosystems, and is therefore considered of economic importance. Its fruits, which weigh on average between 600 and 700 g, have juicy pulp ranging from cream to dark salmon in color, with a sweet almond flavor. In addition to fresh consumption, they are considered medicinal and used in different ways, including seed oil, which is employed for conditions such as asthma, hair loss, and wound healing, as well as its wood, which is valued for its hardness [63,64].

#### 3.2.5. *Manilkara achras*

The name *Manilkara achras* was historically used to designate chicozapote, but it is currently considered a synonym of *M. zapota* [40,46]. Despite this, this scientific name continues to appear in recent publications, especially in Asia, where the species is widely cultivated under the common name “sapota” or “chiku” [53,56,57,65]. The persistence of this term reflects the difficulties in Sapotaceae systematics and the inertia of old or historically used nomenclatures. The most frequent in Asian literature is *Achras zapota*, and other old synonyms are *Lucuma mammosa* or *Calocarpum mammosum* [59].

#### 3.2.6. Other Sapotes of Mesoamerica

In addition to the main Sapotaceae species already mentioned, other fruits known as “sapotes” are recognized in Mesoamerica, although they belong to different families, such as the fruit called black sapote [*Diospyros digyna* (syn. *D. nigra*), Ebenaceae] and white sapote (*Casimiroa edulis*, Rutaceae) [55,66]. Other less studied and less known species of the Sapotaceae family also stand out, such as *Pouteria glomerata*, *Pouteria viridis*, *Sideroxylon capiri*, and *Lucuma* spp.; these are used locally as fruit trees, timber, and/or medicinal plants [2,45,50]. Unlike the former synonyms of *Manilkara zapota*, which are no longer considered valid today, the names mentioned here correspond to the current nomenclature, although some species present historical synonyms. This diversity reflects the biocultural richness of the Mesoamerican region and represents a genetic heritage of great value for food, traditional medicine, and scientific research, whose conservation is strategic in the face of climate change and the growing demand for bioactive compounds [28].

## 4. Morphological and Genetic Characterization

### 4.1. General Morphological Characteristics of the Sapotaceae Family

Species of the Sapotaceae family are characterized as shrubs and trees of pantropical forests. According to Pennington and Sarukhan [55], the family is made up of 540 species, distributed throughout the Southern USA, Mexico, and Central America, including countries such as Paraguay, Chile, and Uruguay. In Mexico, there are 7 genera and 41 species, distributed in the Chiapas, Guerrero, Michoacán, Veracruz, and Yucatán states, in warm (subhumid) and semi-warm (humid) climates, in agroforestry plantations, in the wild, and in commercial plantations.

Among the most important genera for the production of their edible fruits are *Pouteria* with approximately 188 species, Chrysopyllum with 43 species, and *Manilkara* with 30 species [67,68]. These species, in addition to providing fruit, also provide very hard wood and produce a milky sap or chewy white latex [69].

### 4.2. Morphological Characterization of the Main Genera

#### 4.2.1. *Pouteria* Genus

Within the Sapotaceae family, the *Pouteria* genus stands out for its economic and nutritional importance. The following species are among the most studied for their edible fruits.

##### *Pouteria sapota* 

**Figure 3:**
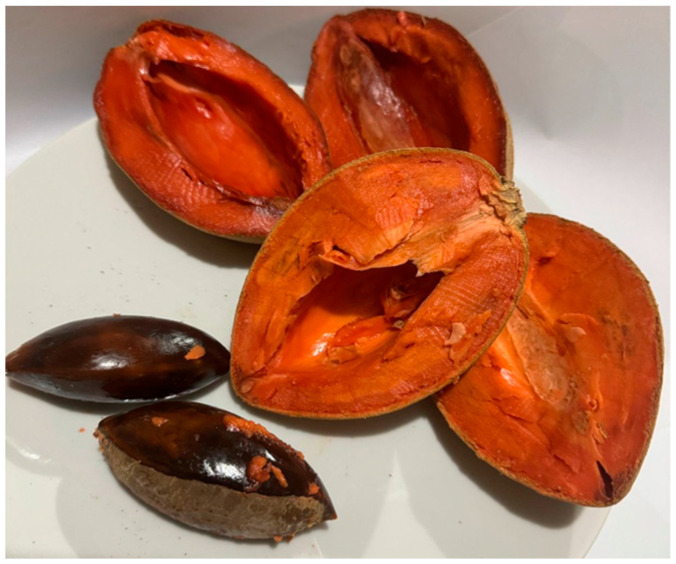
Fruit of *Pouteria sapota* (mamey sapote).

Commonly known as mamey sapote, *P. sapota* is a tropical fruit tree that can reach heights of 12 to 50 m. It has a straight trunk with horizontal, widely spaced branches. Its leaves are oval or lanceolate with obtuse to rounded apices, 10 to 30 cm long and 4 to 10 cm wide [70]. They are dark green on the upper surface and pale green on the underside. Their petioles are glabrous or pubescent, 3–5 cm long. Its flowers are hermaphroditic, actinomorphic, clustered in the leaf axils, forming groups of 3 to 4, of which only one produces fruit [51]. Its calyx is brownish-green with numerous sepals and its corolla, made up of 4 to 5 round or truncated lobes, is greenish-cream in color with 4 to 5 green stamens and brown anthers. The fruit is an ovoid or elliptical monospermous berry with a rough epidermis, with small gray scales that give it a rough texture. The color of the pulp can vary from orange to brown, with 1 to 4 black, ellipsoid seeds (Figure 3) [43].

##### *Pouteria campechiana* 

Known by the common name yellow sapote, *P. campechiana* is a medium-sized, evergreen, monopodial tree that can reach heights of 12 to 20 m. Its leaves are alternate and grouped in whorls at the tips of the branches. They are elliptical and pointed at the apex, 5 to 25 cm long, and glossy green. Its trunk is rich in lumpy white latex. Its flowers are bisexual, with a short pistil, solitary, small (1 cm), white-green, and fragrant, clustered in the axils of leaves or nodes. Its fruits are round with a bulge at the distal end, of variable size, yellow with a thin epicarp, and shiny, dark brown seeds with a rough, light-colored thread on the ventral side. The pulp is sweet, yellow in color and has a texture similar to boiled egg yolk [71,72].

##### *Pouteria viridis* 

Commonly known as green sapote, *P. viridis* is a tree very similar to *P. sapota*, growing up to 40 m tall and 1.5 m in diameter. Its leaves are simple, alternate, cuneate-oblong, 10 to 25 cm long and 5 to 7 cm wide with an obtuse apex, covered with white hairs on the underside. Its flowers form fascicles grouped below the leaf insertion; they are white or pinkish in color, with a five-lobed corolla, five stamens, and five staminodes [73]. Its fruits are berries, 24 cm long and 15 cm in diameter, and come in various shapes, including round, ovoid, or dotted at the apex. Their color is yellowish green, sometimes orange or reddish, with a smooth and shiny texture and, unlike species such as *P. sapota*, they are smaller. The weight of their fruits can vary from 600 to 700 g, and they have brown to light black seeds, smooth, shiny and ellipsoid, from 1 to 2 in small fruits, while in the larger ones there can be up to 4. The color of the pulp varies from creamy to dark salmon tones, and it is characterized by being juicy, sweet, soft and aromatic [74].

#### 4.2.2. *Manilkara* Genus

Commonly known in Mexico as chicozapote, *Manilkara zapota* is an evergreen tree that grows to a large size and can live up to 100 years. It is a slow-growing, polymorphic species that can acquire round or pyramidal shapes. It is formed by a straight, grooved trunk that reaches up to 50 m in height. Branch growth is sympodial, with horizontal basal branches, and the youngest ones have hard, brown, indented trichomes. Their terminal branches are short and have leaves in clusters. A whitish, milky exudate known as chicle (chicle) can be obtained from the grated bark (20–25 cm wide) [75]. It has a vigorous root system that enables it to adapt to water-stress conditions. Its leaves are elliptical and group together at the end of the shoots. When ripe, they are shiny, dark green on the upper surface and opaque on the underside.

The flowers are white, solitary, with greenish-brown sepals, hermaphroditic, whitish bell-shaped and clustered at the tips of the branches. It has a calyx with two whorls of three sepals, six petals, six stamens, and six staminodes [69]. Its fruits are berries measuring 5 to 10 cm in diameter, of various shapes ranging from fusiform to round, with a rough shell, and mesocarp colors ranging from yellow to red or brown. It has long, oval seeds that turn black to dark brown as they ripen [53,59]. This species is widely distributed in Central America and Asia, and has a wide variety of synonyms, including *Manilkara achras*, *Manilkara zapotilla*, *Achras zapota*, *Sapota achras*, among others.

### 4.3. Genetic Characteristics of the Main Genera

#### 4.3.1. *Pouteria* Genus

Plants of the *Pouteria* genus that are propagated by seed through cross-pollination, as occurs in wild, agroforestry, and backyard plants, exhibit greater genetic variability than commercial or grafted varieties, which have undergone selection [50]. Genotypes have been recorded with differences in flowering cycles, ranging from early to late, with flowering periods of 2 to 4 per year, with 12 to 20 flowers per specimen. Fruit ripening periods range from 18 to 21 months, with weight variations between 230 and 850 g, a pulp percentage of 60 to 82 g, and 1 to 2 seeds per fruit [43,67].

According to Aranguren-González and Pérez-Rodríguez [70], the behavior of cultivars varies according to climate and soil conditions, so it is important to carry out plant breeding programs that evaluate the stability and genetic variability of cultivars in different regions. Some studies with microsatellite molecular markers known as SSR (Simple Sequence Repeat) have recently shown a decrease in genetic diversity in wild and cultivated populations in Southeastern Mexico, which may be due to the decrease in genetic flow between these populations [76].

#### 4.3.2. *Manilkara* Genus

Plants of the *Manilkara* genus exhibit great variation in stem or trunk branching morphology, leaf color, fruit shape and size, as well as pulp texture, color, and nutritional quality [77]. However, there are still a few studies on their genetic diversity. SRR molecular markers have been used in *Manilkara* species and have shown greater genetic diversity in species that grow in conserved areas [78]. More recent studies show that this diversity can be reduced by anthropogenic practices, such as the interruption of gene flow between populations, which causes population fragmentation and affects their dispersal and reproduction [79].

Other genetic studies have focused on the detection of expansin genes (*mzexp1* and *mzexp2*), whose proteins form part of the cell wall, loosen the cellular structure to allow the enlargement of plant cells, and these proteins are related to the rapid softening of the fruit. The expression of the *mzexp1* gene was detected during the early stages of development and *mzexp2* at the end of fruit development [80].

## 5. Traditional and Contemporary Uses

**Figure 4:**
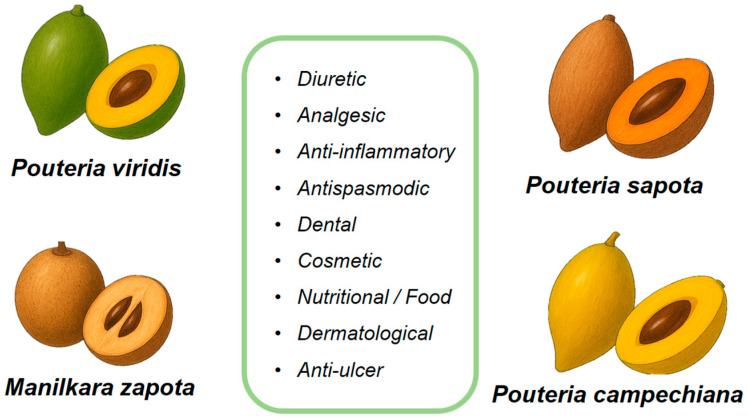
Traditional and ethnobotanical uses of species of the Sapotaceae family.

Species of the Sapotaceae family, native to Mesoamerica, have played a central role in the dietary, medicinal, and cultural practices of various indigenous and rural communities. In addition to their ecological value, these species have significantly contributed to the well-being of local populations through their fruits, seeds, bark, and latex. Here, we provide a summary of the traditional and ethnobotanical uses of four representative species of this family (Figure 4).

### 5.1. Pouteria sapota

Sapota has long been valued for its medicinal properties. The fruits and seeds exhibit diuretic activity, aiding in the prevention of kidney and bladder stones [81], while the fruit latex has been used as a dental filling material. Its anti-inflammatory and analgesic effects contribute to the management of gastritis, reflux esophagitis, and intestinal disorders, with seed pastes traditionally applied to relieve pain and swelling from stings or bites [3], supports intestinal health, strengthens immunity, and protects against bacterial infections; during pregnancy, its high nutrient content helps reduce nausea, dizziness and weakness [82]. Decoctions of the bark and fruit are used to treat fever, diarrhea, and dysentery, and may also alleviate constipation and hemorrhoids. The fiber and vitamin A content are associated with preventive effects against colon, lung, and oral cancers [81]. Additionally, preparations combining sapota flowers and fruits are reported to improve respiratory health, and the fruit itself shows antispasmodic activity [3].

The oil extracted from mamey sapote seeds has been shown to contain a high proportion of oleic acid (approximately 48–53 %) and exhibits a fatty-acid profile similar to other commercial edible oils, suggesting strong oxidative stability and suitability for cosmetic and topical applications [6,83]. While the environmentally friendly enzymatic extraction method ensures a high-quality, solvent-free oil ideal for use in creams, lotions, and other personal care products [84]. In parallel, recent studies have shown that aqueous leaf extracts are rich in phytochemicals and exhibit significant antioxidant and cytotoxic activities, including anti-cancer effects against breast cancer (MCF-7) cell lines. These findings suggest that, beyond its traditional uses, *P. sapota* leaves may hold promising medicinal potential, particularly for conditions related to oxidative stress and cancer [85].

### 5.2. Pouteria campechiana

In Mayan folk medicine, preparations incorporating *Pouteria campechiana* leaves, often combined with *Chrysophyllum cainito*, *Citrus limonum*, and *Annona muricata* in equal proportions, have traditionally been used for pain relief. Pharmacological evaluation of this herbal mixture confirmed its antinociceptive and antihyperalgesic effects, providing scientific support for its ethnomedicinal applications [86]. This species has been traditionally employed to manage inflammation, pain, and peptic ulcers, reflecting its long-standing use in folk medicine. Additionally, the bark has been used for the treatment of fevers and skin conditions, while the seeds have been traditionally applied to manage ulcers. Experimental studies have shown that the ethanolic extracts of *Pouteria campechiana* leaves and seeds exhibit significant antioxidant, antibacterial, and anticancer activities. Specifically, the leaves extract has shown cytotoxic effects against human hepatocellular carcinoma (HepG2) cells, inducing apoptosis through the modulation of reactive oxygen species and key signaling pathways such as ERK1/2, Akt1, JNK1, and VEGFA. The seed extract, on the other hand, has demonstrated potent anticancer activity against HeLa cell lines, with an IC_50_ value of 4 µg/mL, as assessed by MTT and LDH assays. These findings provide scientific support for the ethnomedicinal applications of *Manilkara zapota* [18,87].

### 5.3. Pourteria viridis

Scientific reports on the traditional uses of *Pouteria viridis* are limited. Secondary sources note that its fruit is primarily eaten fresh and, in regions where it is abundant, sometimes transformed into jams [88]. However, detailed ethnobotanical or pharmacological studies validating these uses are scarce.

### 5.4. Manilkara zapota

This fruit is valued for its sweet flavor and versatility, being consumed fresh or processed into sorbets, jams, dried snacks, syrups, vinegar, and juice powders. Its latex has been used as a chewing gum base, and when combined with pectin, the pulp can yield nutrient-rich fruit bars [4]. Traditionally, *Manilkara zapota* has been employed for managing diarrhea, diabetes, dyslipidemia, and obesity-related complications. Its high content of flavonoids, polyphenols, and tannins has been shown to contribute to antioxidant activity, protection against oxidative stress, and antimicrobial potential, supporting its use in medicinal applications and food preservation [53,89,90]. Leaves also exhibit antihyperglycemic, hypocholesterolemic, anti-inflammatory, anti-arthritic, antioxidant, and antibacterial activities, whereas seeds contain diverse secondary metabolites with antimicrobial effects, including activity against *Candida* species, underscoring their therapeutic and nutraceutical potential [86].

## 6. Phytochemical Composition

Fruits from the Sapotaceae family are characterized by their complex nutritional profiles, which include carbohydrates, dietary fiber, proteins, lipids, vitamins, and minerals. Despite their high moisture content (69.46%), mature *Manilkara zapota* fruits offer notable nutritional benefits. The dietary fiber content (5.21–11%) of sapodilla pulp exceeds that found in the pulp of commonly consumed fruits such as mango, pomegranate, and banana. Furthermore, *M. zapota* supplies essential macronutrients, including protein (0.71–1.54%), fat (1.1%), and carbohydrates (21.9%) [8,91].

Interspecific variation in nutritional traits becomes evident upon broader examination of the Sapotaceae family. The pulp of mature *Pouteria campechiana* fruits, for instance, contains higher levels of lipids (5.2%), proteins (4.5%), and carbohydrates (44.1%), while its fiber content remains within a similar range to that of *M. zapota*. However, the nutritional composition of *P. campechiana* is dynamically influenced by the maturity stage. Therefore, as the fruit ripens, the concentrations of fiber and organic acids decline in favor of the accumulation of soluble sugars [5]. The same behavior was observed by Solís-Fuentes et al. [6] in another member of the same genus, *Pouteria sapota*.

The nutritional profile of Sapotaceae fruits is completed by a diverse array of vitamins. The fruit of *Manilkara zapota* contains ascorbic acid, which exhibits the highest con-centration among these micronutrients (approximately 15 mg per 100 g of edible portion) [77,91]. Other vitamins, including riboflavin, niacin, pyridoxine, and pantothenic acid, are also present, though in markedly lower amounts (below 0.2 mg per 100 g). Complementing its vitamin profile, the fruit also harbors various pigments. Silva et al. [7] identified lycopene at a concentration of 41.93 µg per 100 g (dry basis), while Shinwari and Rao [91] documented the presence of carotenoids and anthocyanins, each at 2.5 mg per 100 g.

These constituents contribute to the antioxidant activity reported in Sapotaceae fruits, which was found to surpass that of carrots, bananas, and spinach [8]. Lycopene is recognized for its singlet oxygen-quenching capacity, and L-ascorbic acid plays a prominent role in the antioxidant profiles of many fruits [7]. However, Kaur et al. [92] mentioned that the antioxidant capacity of sapodilla is more accurately attributed to other bioactive phytochemicals, particularly polyphenolic compounds. These and other metabolite classes are distributed throughout the Sapotaceae family, with bioactive compounds extractable not only from edible plant parts but also from non-edible tissues. Qualitative screening revealed that the main categories of phytochemicals isolated from either *P. campechiana* or *M. zapota* are alkaloids, steroids, flavonoids, tannins, phenols, glycosides, saponins, and quinones [10,11].

The abundance and diversity of these phytochemicals, much like the nutrient content previously discussed, are not static; rather, they are shaped by physiological and environmental factors throughout fruit development and handling. As *M. zapota* progresses toward full ripeness (marked by elevated total soluble solids), the levels of polyphenols, including flavonoids and anthocyanins, along with their associated antioxidant activity, tend to decline [93,94]. Agronomic practices and post-harvest handling are critical determinants of both the quantity and bioactivity of these compounds. Costa et al. [93] reported that nitrogen fertilization regimens had an adverse effect on the accumulation of anthocyanins and flavonoids. In contrast, post-harvest strategies, such as the application of malic acid-based coatings, can help preserve the total phenolic content during storage. Notably, combining malic acid with Arabic gum not only retained phenolic levels, but also enhanced flavonoid content and antioxidant activity. These beneficial effects were attributed to reduced polyphenol oxidase activity and increased activity of phenyl-alanine ammonialyase (a key enzyme in flavonoid biosynthesis) [94,95]. Both temperature and storage duration significantly influence polyphenol oxidase activity, thereby modulating the rate of phenolic compound degradation over time [96].

Table 1 provides a broader overview of phytochemicals identified in different segments of Sapotaceae species, highlighting their distinct chemical profiles—flavonoids and phenolic acids in the pulp, triterpenoids in the leaves, and fatty acids in the seeds. These compounds and their derivatives show diverse biological activities, including antioxidant, antimicrobial, anti-inflammatory, anticancer, antihyperglycemic, and antidiabetic effects [12,97].

The data compiled in Table 1 were obtained through targeted search strategies conducted in Scopus. The retrieved documents included the following reference terms in their title, abstract, or keywords, combined with the appropriate Boolean operators. The search queries used were as follows: TITLE-ABS-KEY (Sapotaceae* AND (phytochemicals OR “bioactive compounds” OR polyphenol* OR flavonoid* OR carotenoid*)); TITLE-ABS-KEY ((Pouteria* OR Manilkara*) AND (phytochemicals OR “bioactive compounds” OR polyphenol* OR flavonoids)); TITLE-ABS-KEY (Sapotaceae* AND (phytochemical* OR “bioactive compounds” OR polyphenol* OR flavonoid* OR carotenoid*) AND (“liquid chromatography” OR “gas chromatography” OR “mass spectrometry” OR LC-MS OR GC-MS)).

### Phytochemical Extraction and Recovery Strategies in Sapotaceae

Given the diversity and bioactivity of these phytochemicals, effective extraction strategies are essential to recover target compounds. Isolating compounds of interest from Sapotaceae plants typically starts with drying the raw materials, though directly homogenizing fresh material, such as fruit pulp, is also a common extraction strategy. After being dehydrated at low temperatures or via freeze-drying, the plant material is reduced in particle size to increase its surface area and improve contact with extraction solvents [8,12].

Choosing the right solvent is crucial for maximizing the yield and diversity of recovered compounds. For example, polyphenols, such as flavonoids, are primarily extracted using organic solvents like ethanol, methanol, and acetone, as they have low solubility in water [13,106]. Among these, acetone was reported to yield the most diverse phytochemical profile. However, changing the solvent’s polarity will alter the profile of phytochemicals obtained from a given matrix [10,11]. For instance, solvents like hexane and ethyl acetate lead to isolating a less polar fraction of compounds from *M. zapota* L. P. Roy-en [101].

Extraction yield may be further improved by including repeated extraction cycles, with solvent recirculation and/or continuous mixing. Furthermore, the extraction method can be assisted by technologies designed to promote solvent diffusion and cell wall disruption in plant materials. The extraction of phytochemicals from different segments of Sapotaceae plants has been supported by methods such as ultrasound, high-speed homogenization, and turbolization [12,98,101].

Alternatively, using deep eutectic solvents (DES) or natural eutectic solvents (NADESs) can also achieve high yields while addressing the flammability, poor biodegradability, and toxicity issues associated with conventional solvents [13]. Saha and Chakraborty [99] reported that a DES composed of choline chloride and oxalic acid extracted a higher total content of phenolics, anthocyanins, and flavonoids from *M. zapota* pulp than a mixture of acetone/water. This DES extract mainly contained phenolic acids and flavonoids, with gallic acid, quercetin, protocatechuic acid, and rutin being the primary polyphenols recovered. It is worth mentioning that, although these extracted compounds had a longer half-life, their extraction kinetics were significantly slower.

After extraction, purifying and concentrating the extracts allows for identification and quantification. Solvents are typically removed using a rotary evaporator or vacuum drying [12,101]. Meanwhile, specific compounds can be isolated using processes like solid–liquid extraction, which allows fractionation of compounds based on their polarity. Combining this method with separation techniques like flash chromatography and preparative chromatography yields samples ready for instrumental analysis (mainly gas or liquid chromatography coupled to mass spectrometry) [101].

## 7. Biological Activities

The preservation of the compounds present in these fruits is of great interest, not only for their nutritional value, but also for the set of biological and functional properties attributed to them, mainly related to health and well-being [16,53,102]. Based on this evidence, the following is a summary of the reported findings, organized according to various categories, to highlight the potential of these species and support their importance for study and conservation.

### 7.1. Antioxidant Activity

One of the most consistent pieces of evidence reported for these species is their antioxidant capacity, evaluated through assays such as DPPH, ABTS, and FRAP, as well as the analysis of compounds with redox activity, including phenols, flavonoids, carotenoids, and other bioactive compounds [14]. In particular, the red pulp of *Pouteria sapota* has been shown to possess remarkable antioxidant capacity, which remains constant during the ripening stage, maintaining average IC_50_ values of approximately 12.9 μg/mL [105]. This stability suggests that the antioxidant potential of the fruit does not depend solely on the total phenolic concentration, but also on its qualitative composition. Likewise, it has been reported that hydrophilic extracts have greater antioxidant capacity than lipophilic fractions, with averages of 82.016 ± 4.99 mg AAE/100 g fw in the DPPH assay and 79.290 ± 4.15 mg AAE/100 g fw in the FRAP assay, where AAE refers to ascorbic acid equivalents and fw to fresh weight [107]. Taken together, these results support the antioxidant potential of mamey sapote compounds for health applications.

In *P. campechiana,* various plant tissues such as bark, peel, pulp, seeds, and leaves have been evaluated to determine their antioxidant potential using in vitro assays such as DPPH, ABTS, and FRAP [108,109,110]. Methanolic and ethanolic extracts (70%) were shown to have high antioxidant activity, with IC_50_ values below 50 μg/mL in the DPPH assay. Specifically, the seed extract showed the highest activity (IC_50_ = 0.5 μg/mL), followed by the bark extract. In addition, the authors observed a positive correlation between the total phenol and flavonoid content and antioxidant capacity, suggesting that these compounds are responsible for conferring the biological effect [108,109].

Within the *Pouteria* genus, *P. viridis* has been studied relatively little. However, a study conducted by Ma et al. [103] analyzed the antioxidant capacity of the fruit based on the ethyl acetate fraction of the aqueous methanol extract. Antioxidant activity of IC_50_ 52.6 μg/mL was demonstrated in the DPPH assay, which was positively correlated with its high polyphenolic content. Furthermore, specifically, using liquid chromatography coupled with mass spectrometry (SIM LC-MS), compounds such as gallic acid and (+)-galocatechin had IC_50_ values ranging from 19.0 to 38.3 μM in the DPPH assay, respectively. As with the two previous species, these results confirm their relevance as a natural source of bioactive compounds with antioxidant potential.

The antioxidant capacity of *Manilkara zapota* has been extensively studied in different plant tissues, including fruit, leaves, seeds, and bark, with significant potential reported due to its phytochemical composition [111,112,113]. Among these, the fruit has been shown to have high antioxidant activity, both in ethanolic and aqueous extracts, showing high efficiency in the elimination of free radicals (DPPH and FRAP assays) and a strong correlation with the total content of phenols and flavonoids [111,112]. These extracts showed higher inhibition values than those of the leaves, which is related to a higher concentration of total phenolic compounds and flavonoids [112,113]. In comparative studies, the aqueous fraction of the fruit showed greater reducing capacity and total phenolic content, while seed extracts showed remarkable potential in the ORAC assay [113]. From the compounds isolated from the bark, several flavonoids—such as (+)-dihydrokaempferol and 3,4-dihydroxybenzoic acid—demonstrated high antioxidant capacity in different in vitro evaluation systems [102,113]. Collectively, the evidence supports that *M. zapota* is a promising source of phenolic compounds, and flavonoids with notable antioxidant activity and potential health benefits.

Overall, the evidence suggests that all the species analyzed have a significant antioxidant profile, although differentiated by the predominance of hydrophilic or lipophilic compounds. However, the variability in the methodologies used and the scarcity of in vivo studies limit the comparability of results and the extrapolation to physiological effects. Although, increases in plasma antioxidant capacity have been reported after fruit consumption, more robust research is needed, including more specific clinical studies [114].

### 7.2. Anti-Inflammatory and Immunomodulatory Activity

While antioxidant effects are one of the main lines of defense against oxidative damage, inflammatory and immune system modulation processes represent additional mechanisms that act together as defense and regulation of physiological function.

In the case of *Pouteria sapota*, its phytochemical profile has been characterized by a high concentration of bioactive metabolites with antioxidant, and anti-inflammatory properties [17,105]. These metabolites participate in reducing the expression of proinflammatory mediators such as IL-1β, IL-6, and TNF-α, as well as key enzymes in the inflammatory response, including COX-2 and iNOS [17,115]. Complementarily, the presence of β-carotene in this species has been associated with the suppression of genes related to inflammatory processes and with the activation of antioxidant systems that attenuate oxidative stress, a factor that potentiates chronic inflammatory processes [116,117].

In *Pouteria viridis*, although information is more limited, studies on its phytochemical composition directly evaluate the biological activity of the specie. However, several of the metabolites identified, such as catechin, epicatechin, and myricitrin, have been studied in other plant species, where their ability to regulate gene expression of inflammatory mediators has been observed [103,115]. Therefore, although there is no evidence of the anti-inflammatory effect of *P. viridis*, it is possible to propose an effect based on the presence of these compounds, suggesting therapeutic potential that needs to be explored in future research.

For its part, *Pouteria campechiana* has been studied in relation to the distribution of bioactive compounds in its different tissues. It has been reported that the fruit peel has a higher concentration of phenols and flavonoids compared to the pulp, reflecting superior anti-inflammatory activity using in vitro models [118]. Its phytochemical profile includes flavonoids, lactones, anthocyanidins, and other polyphenolic compounds with the ability to modulate both inflammation and the adaptive immune response [119]. Furthermore, ethanolic seed extracts have been shown to inhibit inflammation by 85% in the carrageenan-induced edema test in animal models. Unlike synthetic non-steroidal anti-inflammatory products, these extracts not only have anti-inflammatory and analgesic effects, but also suggest a safer therapeutic effect. These effects have been linked to their high phenolic and flavonoid content, with protocatechuic acid standing out as one of the metabolites with high inflammation-modulating potential [18,110]. In addition, it has been observed that the methanolic extract of *P. campechiana* leaves exerts a concentration-dependent immunostimulatory effect without compromising cell viability, which supports the consistency of the bioactive effects reported for this species [87].

On the other hand, extracts from *Manilkara zapota* also have remarkable anti-inflammatory and immunomodulatory potential, associated with their secondary metabolites such as alkaloids, polyphenols, and triterpenes [14]. The anti-inflammatory activity has been validated by in vitro and in vivo studies [19,20]. Specifically, crude ethanolic and ethyl acetate extracts from leaves showed significant inhibition of carrageenan-induced edema in rats, an effect associated with the inhibition of inflammatory enzymes such as phospholipase A2 (PLA2) and 5-lipoxygenase (5-LOX). The ethyl acetate extract from the leaves, in particular, exhibited superior activity in inhibiting 5-LOX over inhibiting PLA2 [19]. Complementarily, in another study, it was reported that the ethanolic extract of leaves showed significantly greater anti-inflammatory activity in a murine model of ear edema induced by 12-*O*-tetradecanoylphorbol-13-acetate (TPA). In terms of immunomodulatory activity, the aqueous extract of the unripe fruit regulated proinflammatory cytokines (TNF-α, IFN-γ) and increased IL-10 in mice with type 1 diabetes, promoting the response of regulatory T cells [20].

Overall, the available evidence supports that Sapotaceae species exert their anti-inflammatory activity through mechanisms of inhibition of proinflammatory mediators, regulation of enzymes, and signaling pathways, which, in addition to the antioxidant activity of the tissues, contribute to reducing the risk of oxidative damage associated with inflammatory processes. Given the relevance of these mechanisms, it is interesting to explore other biological activities attributed to these species, in particular their antimicrobial potential, which has been little evaluated.

### 7.3. Antimicrobial and Antifungal Activity

Ethanolic extracts from different tissues of *Pouteria campechiana* have shown antimicrobial activity against bacteria such as *Streptococcus pneumoniae*, *Bacillus subtilis*, and *Escherichia coli*, as well as against fungi such as *Aspergillus fumigatus*, *Syncephalastrum racemosum*, and *Geotricum candidum*. Among the tissues evaluated, the leaf extract stood out for its greater inhibition of *B. subtilis*, while the pericarp extract showed superior antifungal activity against *Syncephalastrum racemosum*. These findings suggest that the presence of bioactive compounds plays an important role in the observed antimicrobial and antifungal properties [120].

*Pouteria*, *P. viridis* and *P. sapota* genera have been less studied, so there are no specific reports on the antimicrobial or antifungal activity of tissue extracts. However, their phytochemical profile, characterized by the presence of polyphenols, flavonoids, triterpenes, and other secondary metabolites, confer them antimicrobial potential, as has been reported in other species [121].

Like the species mentioned above, the antimicrobial and antifungal potential of *Manilkara zapota* is based on its phytochemical profile. It has been reported that the ethyl acetate extract from the stem bark exhibits inhibitory activity against the growth of Gram-positive and Gram-negative bacteria, attributed to the compounds 1-Octanol, 2-butyl-, Tridecane, and 9,17-Octadecadienal (Z) [122,123]. Likewise, lectin has been isolated from its seeds with marked bacteriostatic activity, particularly against Gram-positive bacteria such as *Staphylococcus aureus*, as well as the ability to agglutinate pathogens and inhibit the development of biofilms in *Escherichia coli* [124]. Similarly, fruit extracts and processed foods exhibited greater inhibition of *S. aureus* and *Bacillus subtilis* than Gram-negative bacteria [89]. It has also been noted that extracts from leaves, pericarp, and seeds have moderate to significant activity against various strains, with leaves being the most active tissue [125].

Antifungal activity has also been documented in ethanolic extracts of leaves, showing significant inhibition against *Candida albicans* and moderate action against the dermatophyte *Trichophyton rubrum*, with the flavonoid myricetin-3-*O*-β-D-glucopyranoside being one of the most active compounds [101]. Overall, the antimicrobial and antifungal activity of *M. zapota* is attributed to metabolites such as flavonoids, tannins, quinones, terpenoids, and glycosides, which support its potential as a source of bioactive compounds of pharmacological interest [101].

## 8. Nutraceutical and Industrial Applications

Fruits represent a group of resources that are appreciated by consumers thanks to their flavor and taste characteristics, in addition to the phytochemical profile of both their edible parts and fruit parts that are usually discarded, such as peel, seeds, and pomace. This has generated growing interest in their potential contribution of bioactive compounds, natural molecules, and beneficial properties [126]. In this context, fruits belonging to the Sapotaceae family, and in particular species such as *P. sapota* (mamey sapote), *P. campechiana* (canistel), *P. viridis* (green sapote), and *M. zapota* (chicozapote), have a little-explored profile but potential for application in both the food and pharmaceutical industries [15].

In this sense, the value of these fruits lies not only in their composition, but also in the various ways in which they can be integrated into functional food products and formulations. Currently, the growing demand for products that contribute to general well-being, together with therapies for metabolic, cardiovascular, digestive, or neurodegenerative disorders, has driven research aimed at designing new solutions for human health [127,128].

### 8.1. Applications in the Food Industry

The food sector is one of the main areas benefiting from the use of functional ingredients derived from tropical fruits or their by-products for the development of nutraceutical products [129]. The use of Sapotaceae species responds to the market’s need for formulations and products that combine nutritional quality, desirable organoleptic qualities, and nutraceutical properties, which is why this interest has resulted in various applications [130,131].

#### 8.1.1. Development of Functional Foods

Functional foods have established themselves as a rapidly expanding sector, particularly in markets where consumers are looking for natural alternatives with a focus on sustainability [132,133,134]. In this context, *P. campechiana* has been explored as an ingredient in functional beverages, in combination with guava pulp (*Psidium guajava* L.) and stevia leaves (*Stevia rebaudiana*). The result was a beverage with bioactivity capable of facilitating the conversion of β-carotene to retinol, confirming its value as a dietary source of provitamin A [135].

The fruit has also been processed into powder form for incorporation into cold coffee formulations. Combining the fruit powder with coffee beans produced a beverage with good sensory acceptance and helped to mitigate the intense flavor of ripe *P. campechiana* fruits [136]. Similarly, dried pulp flour has been used in an egg-based mousse ice cream. The incorporation of 0.4% *w*/*w* improved its functional value by increasing antioxidant and antimicrobial activity, as well as improving the color, texture, and stability of the product, making it a healthy and antioxidant-rich alternative [137].

Various species of the Sapotaceae family share comparable applications. *M. zapota* has been used in ice cream, with improvements in physicochemical and functional properties, antioxidant activity, and total dissolved solids content [138]. In addition, High-Pressure Processing (HPP) was used to produce a low-calorie jam, reducing sugar content by 47% and improving texture and rheological properties thanks to fiber, achieving greater acceptability compared to commercial jam [91]. Complementarily, it has been shown that the cut size of the fruit influences the drying and sensory characteristics of the tea, including acidity, bitterness, and astringency, which are associated with the presence of polyphenols [139]. Moreover, in dairy products, Madhubhashini et al. [140] reported that the addition of *M. zapota* pulp to curdled yogurt improved the sensory and nutritional profile, as well as microbiological stability. On the other hand, Konfo et al. [141] developed a beverage based on *M. zapota* puree and pineapple juice, in which the combination with lemongrass essential oil improved sensory acceptance and reinforced its potential as a functional ingredient in new beverages.

#### 8.1.2. Ingredient Substitution

The valorization of tropical fruits also implies their potential for ingredient substitution, including synthetic ingredients such as colorants that cause uncertainty among consumers regarding their safety and sustainability [142]. The case of *P. campechiana* is relevant due to its high content of carotenoids, compounds with antioxidant activity, precursors of vitamin A, and of interest in foods, supplements, and drugs [143,144]. Beyond additives, the use of fruit flour as partial substitute in baking and other products has also been explored. Flours derived from Sapotaceae species have been used as texture enhancers and sources of dietary fiber, contributing to digestive benefits. In muffins, *P. campechiana* flour was used as a partial substitute for wheat flour, modifying the carbohydrate and energy content, improving their physicochemical properties, and reducing the gluten content [145]. Complementarily, concentrated fiber powder was used to enrich cookies. The formulation with 7% concentrated powder had greater sensory acceptance, showing its value as a healthy alternative in baked goods [146].

#### 8.1.3. Food Preservation

Finally, food preservation represents another line of application, and various studies have explored the use of *P. campechiana* to produce films as a strategy to extend the shelf life of products. *P. campechiana* pericarp waste has been transformed into edible films for the preservation of tilapia meat [147] and duck breast [148], inhibiting microorganisms and thus helping to increase the storage time of the meat. The safety of the film has also been demonstrated, with no toxicity reported in murine models [147].

### 8.2. Applications in the Pharmaceutical Industry and Human Health

Interest in the Sapotaceae family also extends to other sectors such as the pharmaceutical industry, mainly due to the phytochemical richness that characterizes its fruits, leaves, bark, and seeds, which have demonstrated biological activities thanks to a wide variety of bioactive compounds with therapeutic potential [53].

#### 8.2.1. Potential Against Metabolic Diseases

Diabetes is one of the most important diseases worldwide, and the search for natural alternatives for its treatment has involved the use of plant species, including fruit trees as a source of extracts with beneficial properties [149]. According to recent research, *P. campechiana* and *M. zapota* exhibit antidiabetic and hypolipidemic activity. Methanolic extracts from the leaves and fruits of *P. campechiana* (Kunth) Baehni showed antidiabetic effects by inhibiting the α-amylase and α-glucosidase enzymes, and also induced the uptake of 2-NBDG (2-(N-(7-nitrobenz-2-oxa-1,3-diazol-4-yl)amino)-2-deoxyglucose), a glucose analogue [150].

With regard to *M. zapota*, the ethanolic extract of the peel and its ethyl acetate fractions showed antihyperglycemic and hypolipidemic effects in murine models, in addition to attenuating complications associated with the disease, such as restoring renal balance, reducing cardiovascular risk markers, and improving peripheral neuropathy [151]. Similarly, the ethanolic extract of the stem bark produced α-glucosidase inhibition and antihyperglycemic effects, improved cardiovascular risk indices, and preserved renal architecture according to histological studies [152]. In Wistar rats, consumption of the leaves and pulp of the fruit significantly improved glucose, insulin, leptin, total cholesterol, and triglyceride concentrations, as well as promoting an increase in HDL cholesterol and limiting body weight gain [9]. In addition, the ethanolic leaf extract modulates lipid metabolism, promoting changes in parameters such as increased HDL through the inhibition of the enzyme HMG-CoA reductase, attributed to the antioxidant capacity of flavonoids. Likewise, the presence of alkaloids inhibits pancreatic lipase, which increases the secretion of lipids in the feces [153].

#### 8.2.2. Applications in Pain and Inflammation Management

Trials in animal models have confirmed that the use of Sapotaceae species provides pain relief. Ethyl acetate extract from *M. zapota* leaves has been shown to have anti-inflammatory and anti-arthritic activities, due to the reduction of edema in rat paws, an effect comparable to that of diclofenac, associated with the inhibition of phospholipase A (sPLA2) and 5-lipoxygenase (5-LOX). In terms of anti-arthritic activity, it reduces the risk of protein denaturation [19]. In addition, prenylated coumarins from the fruits, such as Manizapotina A, can inhibit nitric oxide production in RAW 264.7 mouse macrophages, an effect superior to that produced by hydrocortisone. These anti-inflammatory effects are associated with the presence of flavonoids, phenols, tannins, terpenoids, and steroids [154].

#### 8.2.3. Anti-Infective Agents: Antimicrobial, Antifungal, and Antiviral

The increase of bacterial resistance, with a global impact, has led to the exploration of natural products as possible antimicrobial alternatives [155]. The ethanolic extract of *M. zapota* fruits was evaluated in antimicrobial assays, showing antimicrobial activity against Gram-positive (*Staphylococcus aureus*, *Micrococcus luteus*, *Listeria monocytogenes*, and *Bacillus cereus*) and Gram-negative (*Klebsiella pneumoniae*, *Pseudomonas aeruginosa*, and *Escherichia coli*) bacteria [21]. However, it was also shown that combining the hydroethanolic extract of the leaves with clinical antibiotics such as ciprofloxacin and levofloxacin produces an antagonistic effect and decreases the biocidal level of the antibiotic [155]. The antimicrobial effect is attributed to the action of tannins, flavonoids, and polyphenols, which cause membrane disruption, iron chelation, and inhibition of DNA/RNA synthesis [21].

An alternative is the synthesis of silver nanoparticles (AgNPs) from *M. zapota* fruit extract. Their incorporation into topical gels exhibited antimicrobial activity against pathogenic bacteria such as *S. aureus*, *E. coli*, *P. aeruginosa*, *B. subtilis*, and fungi *Aspergillus niger* and *Candida albicans*, comparable to or superior to the controls [156]. Similarly, Ayodhya et al. [157] reported that CeO_2_ nanoparticles stabilized with fruit peel extract have antimicrobial and antifungal activity, the efficacy of which depends on the concentration.

#### 8.2.4. Neurological Health and Neuroprotection

The fruits of *P. campechiana* and *M. zapota* are a source of micronutrients and compounds that help modulate the epigenetic program essential for neuronal health [22,158]. Ethanolic extracts and powder from the dried fruit of *P. campechiana* (Kunth) Baehni showed neuroprotective effects in an Alzheimer’s model induced in rats. The effect was associated with behavioral improvements, increased total protein content, glutathione reductase, acetylcholinesterase, and catalase, as well as increased malondialdehyde content [22]. In contrast, the ethanolic extract of the *M. zapota* leaf showed a depressant action on the central nervous system (CNS) in mice, prolonging the sleep time induced by phenobarbital [53].

As described by Russo et al. [158], the neuroprotective effects are due to the content of vitamins (A, C, and B complex) and polyphenols, which regulate DNA methylation and histone modifications, promoting neuronal differentiation, synaptic plasticity, and the prevention of neurodegeneration such as Alzheimer’s disease. This is mainly due to its antioxidant, anti-inflammatory, and epigenetic enzyme-modulating actions.

#### 8.2.5. Anticancer and Cytotoxic Activity

*P. campechiana* and *M. zapota* exhibit cytotoxic activity against several cancer cell lines. Pramod et al. [159] analyzed the effect of the ethanolic extract of *P. campechiana* bark extract on cell lines and confirmed a dose-dependent degree of cytotoxicity for Ehrlich ascites carcinoma (EAC) and Dalton lymphoma ascites (DLA), as well as the inhibition of cell proliferation in the Human Cervical Cancer (HeLa) line, which may be associated with a mechanism of action with antitumor properties.

For its part, *M. zapota* has shown similar cytotoxic activity. According to Rani et al. [160], the hydroalcoholic extract of the fruit showed significant activity against the A431 skin cancer cell line. In addition, molecular docking studies observed a high affinity of quercetin for the structure of skin cancer, suggesting a possible direct interaction with key proteins in this type of cancer.

This shows that Sapotaceae species, thanks to their phytochemical characteristics, stand out as valuable sources for the development of functional foods and as alternatives for the prevention or management of various diseases.

## 9. Future Research Perspectives

The Sapotaceae family is characterized to have 1250 species [15], with at least 53 genera [161]; this family is widely distributed in tropical areas around the world. Some of the most representative species of this family are *Chrysophyllum cainto*, *Madhuca longifolia*, *Pouteria mammosa*, *Manilkara zapota* and *Mimusops elengi* [161], *Pouteria caimito* [162,163], *Manilkara zapota* [53], *Sideroxylon lanuginosum* Michaux [164], and *Sideroxylon cinereum* [165]. However, despite this extended distribution, the chemistry, nutritional content, and biological activities of most of these species remain relatively unexplored [15]; the most studied *Sapotaceae* species are those with economic potential because of their edible fruits [27].

Some of the parts of these plants most studied for their chemical composition and the biological activities of their phytochemicals are the peel, pulp, seed [162], tree bark and leaves [53]. The content of phytochemicals varies depending on the part of the plant used for its extraction. de Oliveira et al. [162] report that the highest activities of phenolic compounds are in the fruit peel followed by the pulp and seeds of some species of Sapotaceae family. The seeds have purgative and diuretic activities [53]. Other parts that have been used as sources of phytochemicals from these species are tree bark, which has antibiotic, antidiarrheal, astringent, and antipyretic activities, and leaves that are traditionally used to treat diarrhea, cold, indigestion, cough, hemorrhages, fever, ulcers, and wounds [53]. The use of edible fruit pulp for the isolation of bioactive compounds impacts producers economically and reduces food availability. Depending on the plant’s phenological stage, the use of leaves for compound extraction can affect fruit production and quality, as well as the accumulation of reserves in the stem and root for the following year’s production. Furthermore, the use of tree bark could affect tree health, as it could cause injury and provide an opportunity for pests or pathogens to enter or infect the tree.

Through traditional medicine or by the bioactive compounds identified in the species of the Sapotaceae family, these have been associated with different biological activities such as: antioxidant, cytotoxic [162], anti-inflammatory, antibacterial, antifungal, antiulcer [14,27], anticariogenic, anti-pancreatic lipase [166], anti-dental caries, anti-obesity [166], anti-chicken pancreatic lipase [166], anti-α-glucosidase, anti-α-amylase, antiproliferative activity against HeLa, HT-29 and MCF-7 cancer cell lines [164], anti-diabetic [161,167] anti-diarrhea, anti-cough, anti-fever and laxative effects [163].

The number and quantity of bioactive compounds extracted from a plant sample will depend, among other things, on the solvent used and the extraction method. When hexane was used as a solvent, 19 compounds were identified, including volatiles and fatty acids (palmitic, elaidic, and linoleic acids), while with methanol, 17 compounds (catechin and its derivatives) were identified [162]. This will also affect the biological activity of the extract or the extracted compounds. Bhajan et al. [165] indicate that antioxidant activity depended on the organic solvent and the part of the fruit used, while antimicrobial activity was affected by the solvent used. Ethyl acetate extract showed an anti-bacterial effect (*E. coli*, *S. epidermidis*, *S. aureus*, *Klebsiella pneumonie*), but not for *Enterococcus faecalis* and *Salmonella* tiphy [163]. While methanol extract promoted a dose-dependent inhibition of *Streptomyces mutans* isolates [166], the climatic and soil conditions of the place where the plant grows also have a very important effect on the bioactivity of phytochemicals. Brintha et al. [168] evaluated the antimicrobial activity of *Manilkara zapota* plants produced in two different locations, with plants from site II showing the greatest activity against *Bacillus subtilis* and *Aspergillus flavus*. On the other hand, in most studies of bioactive compounds with plants of the Sapotaceae family, conventional extraction methods have been used (maceration, percolation, infusion, soxlet, etc.), and have mainly used organic solvents that could have negative effects on the environment; for example, Brintha et al. [168] used maceration and organic solvents (petroleum ether, ethyl acetate, chloroform, methanol, propanol, and acetone). Therefore, it is urgent to use more environmentally friendly extraction methods for bioactive compounds (alternative technologies, v.g., ultrasound, microwaves, supercritical fluids, etc., or combinations of these alternative technologies), as well as switching to “green” solvents, v.g., water, ethanol, deep euteric solvents.

From plants of the Sapotaceae family, extracts have been obtained containing compounds with applications in the food, pharmaceutical, and textile industries, among others. Some of these compounds are: saponins, flavonoids [14], tannins, alkaloids, terpenoids [166], triterpenoids, saponins [169] quercetin glucoside [164] phenolic acid, other non-flavonoid phenolics [167], triterpenoid saponins [161], lanosterol acetate [165], sugars, proteins, amino acids, and minerals [53]. However, it is considered that there are still many bioactive compounds to be identified and isolated and their biological activities tested from plants of the Sapotaceae family, if alternative technologies and a wider range of solvents, especially “green” solvents, were used [27].

The bioactive compounds extracted from plants from the Sapotaceae family have great potential to be used in different industries; however, there are still a lot of research gaps, especially in species of this family that have inedible fruits. All of the above serves the purpose of identifying new compounds that can be used as new drugs [169], so it is important to determine the biological activities of these compounds, using both *in vivo* and human studies, to identify their negative effects and their mechanisms of action [167].

## 10. Conclusions

The *Pouteria sapota*, *Pouteria campechiana*, *Pouteria viridis*, and *Manilkara zapota* species are fruit resources of high biological, cultural, and economic value in Mesoamerica. The scientific evidence reviewed in this article confirms that these species are important sources of bioactive compounds with significant potential for the prevention of chronic diseases, as their biological activities, including antioxidant, anti-inflammatory, antimicrobial, and anticancer effects, have been reported. Nevertheless, gaps remain in the literature concerning the standardization of extraction methods, the elucidation of mechanisms of action, and validation through *in vivo* and clinical models—factors that are essential to support their application in the food and therapeutic industries. Therefore, it is necessary to foster multidisciplinary research integrating chemistry, biotechnology, and health sciences, intending to promote the sustainable use of these species and their nutraceutical and therapeutic potential, while also contributing to the development of innovative products that benefit both human health and producing communities.

## Figures and Tables

**Table 1:** Compounds isolated from different species of Sapotaceae family.

Species	Plant Segment	Phytochemicals	Analytical Technique	References
*Manilkara zapota*	Fruit pulp	Flavonoids Rutin, catechin, quercetin and kaempferol. Phenolic acids Gallic acid, protocatechuic acid, vanillic acid, chlorogenic acid, caffeic acid, *p*-coumaric acid, ferulic acid. Carbohydrates and derivatives α-D-glucopyranose, α-L-ramnopyranose, L-galactose, L-lyxose, xylose, sucrose, 3-deoxy-D-mannonic acid, 3-deoxy-D-mannoic lactone, inositol-L-dexy, muccic acid. Fatty acids and derivatives Dodecanoic acid, n-hexadecanoic acid, 9,12-octadecanoic acid. Various Amyrin, trioxsalen, thymine, tryptamine, diisooctyl phthalate.	GC-MS; HPLC	[98,99]
	Fruit peel	Phenolic acids Gallic acid. Flavonoids Catechin, quercetin, kaempferol. Fatty acids and derivatives Palmitic acid, heptadecanoic acid, stearic acid, oleic acid, linoleic acid, arachidic acid, linolenic acid	HPLC-DAD; GC-FID	[8,100]
	Leaves	Flavonoids and derivatives Afzelechin, epicatechin, epigallocatechin, myricetin, ampelopsin, laricitrin, myricetin-3-*O*-rhamnoside, laricitrin-3-*O*-rhamnoside, myricetin-3-*O*-β-D-glucopyranoside, mearnsetin-3-*O*-α-L-rhamnopyranoside, apigenin-7-*O*-β-D-glucuronide methyl ester, leucodelphinidin. Phenolic acids and derivatives 3,4-dihydroxybenzoic acid, salicylic acid, vanillic acid, ferulic acid, syringic acid, gallic acid, caffeic acid, chlorogenic acid, 3-*O*-galloylquinic acid, 3-glucogallic acid, 3-*p*-coumaroylquinic acid. Triterpenoids and derivatives Germanicol, β-amyrin, α-amyrin, germanicol acetate, α-amyrin acetate, lupeol acetate. Various 2-hydroxybenzaldehyde, threonic acid, pyroglutamic acid, esculetin, hydroquinone glucuronide.	APCI/ESI-MS/MS; LC-MS; TLC-UV; GC-MS; HPLC-DAD/CAD; NMR	[12,19,101]
	Seeds	Fatty acids and derivatives Palmitic acid, oleic acid, heptadecanoic acid, stearic acid, linoleic acid, arachidic acid, linolenic acid, myristic acid, pentadecanoic acid, glycidol stearate, 15-hydroxypentadecanoic acid, (9*E*)-9-octadecenoic acid, methyl hexadecanoate. Hydrocarbons 1-hexadecene, 1-octadecene octadecane, nonadecane, dodecane, n-tetradecane. Various Phenol,2,4-bis(1,1-dimethylethyl), dibutyl phthalate, 5-oxotetrahydro-2-furancarboxylic acid.	TLC-UV; GC-MS; GC-FID	[10,100]
	Bark	Flavonoids 6-Hydroxyflavanone, (+)-Dihydrokaempferol Phenolic acids 3,4-Dihydroxybenzoic acid Triterpenoids and derivatives Taraxerol methyl ether, taraxerol, taraxerone, lupeol acetate. Sterols Spinasterol	TLC-UV; HR-ESI-MS; NMR; HPLC-UV	[102]
*Pouteria campechiana*	Whole fruit	Phenolic acids Gallic acid. Flavonoids and derivatives (+)-Catechin, (+)-gallocatechin, myricitrin	TLC; LC-ESI-MS; NMR	[103]
	Leaf	Flavonoids Gallocatechin, catechin, epicatechin, dihydromyricetin. Hydrocarbons Octacosane, hexatriacontane, tetracosane.	GC-MS; FTIR; TLC-UV; NMR	[11]
*Pouteria sapota*	Fruit pulp	Terpenes and terpenoids α-pinene, 4-terpineol, α-cedrene, thujopsene, β-cadinene, δ-cadinene, β-ionone, cedrol, dihydroactinidiolide, cadalene. Fatty acid esters Methyl isomyristate, methyl myristate, methyl palmitate, ethyl palmitate, isopropyl palmitate, methyl oleate. Various Benzaldehyde, naphthalene, azulene, benzophenone. Phenolic acids Gallic acid Protocatechuic acid p-hydroxybenzoic acid Flavonoids Epicatechin Catechin Rutin	HS-SPME-GC-MS HPLC-DAD-MS	[17,104,105]
*Pouteria viridis*	Whole fruit	Phenolic acids Gallic acid. Flavonoids and derivatives (+)-Catechin-3-*O*-gallate, (+)-catechin, (−)-epicatechin, (+)-gallocatechin, dihydromyricetin, myricitrin.	TLC; LC-ESI-MS; NMR	[103]
